# Integration of cancer stemness and neoantigen load to predict responsiveness to anti-PD1/PDL1 therapy

**DOI:** 10.3389/fcell.2022.1003656

**Published:** 2022-11-17

**Authors:** Kunpeng Luo, Shuqiang Liu, Xiuyun Shen, Jincheng Xu, Chunpeng Shi, Yuqiu Chao, Zhengchao Wen, Kejiao Zhang, Ru Wang, Bing Liu, Yanan Jiang

**Affiliations:** ^1^ Department of Pharmacology State-Province Key Laboratories of Biomedicine-Pharmaceutics of China, Key Laboratory of Cardiovascular Research, Ministry of Education, College of Pharmacy, Harbin Medical University, Harbin, China; ^2^ Department of Gastroenterology and Hepatology, The Second Affiliated Hospital of Harbin Medical University, Harbin, China; ^3^ Department of Hepatopancreatobiliary Surgery, The Second Affiliated Hospital of Harbin Medical University, Harbin, China; ^4^ College of Basic Medicine, Harbin Medical University, Harbin, China; ^5^ Department of Oral and Maxillofacial Surgery, The First Affiliated Hospital of Harbin Medical University, Harbin, China; ^6^ Translational Medicine Research and Cooperation Center of Northern China, Heilongjiang Academy of Medical Sciences, Harbin, China

**Keywords:** tumor neoantigen burden, stemness, immunotherapy, drug resistance, prognostic model

## Abstract

**Background:** Anti-programmed cell death 1/programmed cell death ligand 1 (PD1/PDL1) therapy is an important part of comprehensive cancer therapy. However, many patients suffer from non-response to therapy. Tumor neoantigen burden (TNB) and cancer stemness play essential roles in the responsiveness to therapy. Therefore, the identification of drug candidates for anti-PD1/PDL1 therapy remains an unmet need.

**Methods:** Three anti-PD1/PDL1 therapy cohorts were obtained from GEO database and published literatures. Cancer immune characteristics were analyzed using CIBERSORTX, GSVA, and ESTIMATE. WGCNA was employed to identify the gene modules correlated with cancer TNB and stemness. A machine-learning method was used to construct the immunotherapy resistance score (TSIRS). Pharmacogenomic analysis was conducted to explore the potential alternative drugs for anti-PD1/PDL1 therapy resistant patients. CCK-8 assay, EdU assay and wound healing assay were used to validate the effect of the predicted drug on cancer cells.

**Results:** The therapy response and non-response cancer groups have different microenvironment features. TSIRS was developed based on tumor neoantigen and stemness. TSIRS can effectively predict the outcomes of patients with anti-PD1/PDL1 therapy in training, validation and meta cohorts. Meanwhile, TSIRS can reflect the characteristics of tumor microenvironment during anti-PD1/PDL1 therapy. PF-4708671 is identified as a potential alternative drug for patients with resistance to anti-PD1/PDL1 therapy. It possesses significant inhibitive effect on the proliferation and migration of BGC-823 cells.

**Conclusion:** TSIRS is an effective tool in the identification of candidate patients who will be benefit from anti-PD1/PDL1 therapy. Small molecule drug PF-4708671 has the potential to be used in anti-PD1/PDL1 therapy resistant patients.

## Introduction

In recent years, the clinical application of immunotherapies, especially immune checkpoint inhibitors, has brought revolutionary advancements in the comprehensive cancer therapy (Yang, 2015; Riley et al., 2019; He and Xu, 2020). Programmed cell death 1/programmed cell death ligand 1 (PD1/PDL1) axis plays an important role in tumor progression and immune surveillance evasion (Chamoto et al., 2017; Iwai et al., 2017). CD8 T cells are the major conductor of cancer cell-eliminating (Chen and Mellman, 2013). In the immune microenvironment, the interaction of PD1 and its ligand PDL1 inhibits the activation of CD8 T cells and promotes cancer to evade from the immune mediate tumor elimination. Anti-PD1/PDL1 treatment is the therapeutic strategy that targets cancer immune evasion (Marin-Acevedo et al., 2018). Anti-PD1/PDL1 drugs such as nivolumab, pembrolizumab and atezolizumab were proved by Food and Drug Administration (FDA) in the treatment of solid tumor and brought long-term clinical benefits to patients (Robert et al., 2015; Hodi et al., 2018; Schmid et al., 2018; Larkin et al., 2019). However, the high non-response rate to PD1/PDL1 blockade remains a problem in the clinical practice (Poznanski et al., 2021). Meanwhile, a non-negligible proportion of patients will develop drug resistance and suffer from the side effect of anti-PD1/PDL1 therapy (Horn et al., 2017; Anagnostou et al., 2019). These disadvantages of PD1/PDL1 blockade limited its clinical application. Thus, there is an urgent need to identify the candidates who will benefit from anti-PD1/PDL1 therapy.

The tumor immune microenvironment plays a pivotal role in anti-PD1/PDL1 therapy (Lei et al., 2020). The heterogeneity of tumor immune microenvironment is the major mechanism of resistance to anti-PD1/PDL1 therapy (Lei et al., 2020). According to the microenvironment characteristics and the potential immunotherapy responsiveness, Chen DS et al. divided the cancer immune microenvironment into three subtypes: “immune inflamed”, “immune excluded”, and “immune desert” (Chen and Mellman, 2017). Similarly, Duan Q et al. classified tumors into hot tumors and cold tumors based on the immune infiltration status to predict the clinical efficiency of immunotherapy (Duan et al., 2020). Therefore, revealing the characteristics of tumor microenvironment is important for the rational use of anti-PD1/PDL1 agents.

Cancer stemness can reflect the potential of cancer in self-renewal and dedifferentiation (Gasch et al., 2017). This characteristic was originally derived from the normal stem cells that possess the ability to develop into all cell types (Takahashi and Yamanaka, 2006). As cancer progress, cancer cells will gradually change their differentiated phenotype and obtain progenitor-like and stem-cell-like characteristics (Friedmann-Morvinski and Verma, 2014; Ge et al., 2017). These dedifferentiation related features of cancer play essential roles in the cancer distant metastasis and are potential therapeutic targets for immunotherapy (Chen et al., 2021; Unver, 2021). In 2018, Malta et al. developed mRNAsi, a robust cancer stemness calculating tool based on the PCBC dataset, which provides an opportunity to evaluate the stemness of solid tumors (Malta et al., 2018).

Tumor neoantigen burden (TNB) is also an effective indicator in predicting the responsiveness of patients during anti-PD1/PDL1 therapy (Wang J. Q. et al., 2021). TNB is defined as the number of neoantigens per MB in the genome region (Wang et al., 2019). Since not all antigens caused by somatic mutations can be processed, presented, and recognized by T cells, TNB is considered as a better biomarker than tumor mutation burden (TMB) for predicting the efficiency of anti-PD1/PDL1 therapy (Coulie et al., 2014). Patient-specific neoantigens that generated by tumor-specific mutations are a major factor affecting the efficiency of clinical immunotherapy (Schumacher and Schreiber, 2015). For example, high TNB predicts a better prognosis for non-small cell lung cancer in anti-PD1/PDL1 therapy (Rizvi et al., 2015). Meanwhile, TNB also performs well in predicting responsiveness of other immunotherapy strategies, including anti-CTLA4 therapy and adoptive T cell therapy (Van Allen et al., 2015; Lauss et al., 2017). TMB reflects cancer mutation frequency, while TNB specifically reflect the mutations resulting in the production of neo-antigens which can be presented to T cells (Jardim et al., 2021). Thus, TNB is an effective hallmark in predicting cancer immune microenvironment status and the potential for immunotherapy response (McGranahan et al., 2016).

The rapid development of big data provides us the opportunity to apply the machine-learning methods to promote clinical precision therapy. In order to facilitate individualized anti-PD1/PDL1 therapy, our study aims to predict cancer immune characteristics and patient prognosis during anti-PD1/PDL1 therapy by integrating cancer stemness and TNB features. In this study, we first obtained the multi-omics data and clinic information from several anti-PD1/PDL1 therapy cohorts. Next, we integrated the characteristics of cancer stemness and cancer TNB, and conducted WGCNA analysis to identify the module correlated with these characteristics. Subsequently, the tumor neoantigen and stemness based immunotherapy resistance score (TSIRS) was constructed using machine learning methods to predict the prognosis of patients with anti-PD1/PDL1 therapy. The TSIRS was validated in multiple validation cohorts. We then analyzed the therapy response related microenvironment heterogeneity and the correlation between immune features and TSIRS. Finally, we explored the clinical application potential of PF-4708671.

## Methods

### Data acquisition

In the training cohort, multi-omics data (including TNB and TMB) and clinic information (including prognosis, immune subtypes, and IC subtypes) of metastatic urothelial cancer immunotherapy cohort-IMvigor210 were obtained based on the R package “IMvigor210CoreBiologies” (Mariathasan et al., 2018). IMvigor210 cohort was derived from the study by Sanjeev Mariathasan et al. (Mariathasan et al., 2018). Samples were divided into different IC subtypes and immune subtypes. IC subtypes were divided according to the PD-L1 expression on immune cells assessed by SP142 immunohistochemistry assay. IC0: <1%, IC1:≥1% and <5%, IC2+≥5%. Immune subtypes were divided based on the tumor microenvironment status. Inflamed and IC2+ subtypes have the best immunotherapy responsiveness, while desert and IC0 subtypes have the worst immunotherapy responsiveness in their respective classifications. As for the two validation cohorts, transcriptome data and clinic information of GSE91061 cohort (melanoma immunotherapy cohort) were acquired from GEO. The clinical information and transcriptome data of David A. Braun cohort (clear cell renal cell carcinoma immunotherapy cohort) were obtained from the work of David A. Braun et al. (Braun et al., 2020). Meta cohort was constructed by R package “SVA”. To evaluate cancer stemness, the gene expression profiles of stem cells were acquired from the Progenitor Cell Biology Consortium (PCBC, https://progenitorcells.org/) *via* R package “synapser”.

### Cancer immune microenvironment analysis

To calculate the infiltration level of the immune cells, we applied the deconvolution algorithm “CIBERSORTx” by CIBERSORTx (https://cibersortx.stanford.edu/) (Newman et al., 2019). The immune score, stromal score and ESTIMATE score were calculated *via* the algorithm “ESTIMATE”. Detailed information of cancer immune circle related pathways was derived from the database TIP (Xu et al., 2018). Cancer immune circle related pathway activity was measured by algorithm “GSVA” (Hänzelmann et al., 2013).

### Cancer stemness analysis

The one-class logistic regression (OCLR) algorithm based on the gene expression profiles of stem cells *via* R package “glmnet” was used to estimate the stemness of cancer (Engebretsen and Bohlin, 2019). Detailed process of the stemness index calculation was conducted according to the research of et al. (Malta et al., 2018). The stemness index generated by the algorithm was defined as mRNAsi.

### Weighted correlation network analysis

To identify the co-expressed gene modules related to the cancer stemness and the tumor neoantigen load, we applied the R package “WGCNA” to construct the gene co-expression network (Langfelder and Horvath, 2008). The soft-threshold β value of the co-expression network was selected as 5. Adjacency matrix was transformed into the topological overlap matrix (TOM). MinModuleSize was set as 100.

### Enrichment analysis

Enrichment analysis was applied to analyze the biology function mediated by the module component genes, the. Metascape (https://metascape.org/) was used to analyze and visualize the result of the enrichment analysis (Zhou et al., 2019).

### Construction of the model to predict immunotherapy responsiveness

To recognize the gene modules significantly correlated with cancer neoantigen load and cancer stemness, we analyzed the correlation between gene module and TNB, mRNAsi, TMB, therapy responsiveness, respectively. Genes of modules that significantly correlated with the above features were selected. Then, the stepwise Cox model was implemented for the further gene selection. Stepwise algorithm was conducted in the AIC (Akaike information criterion) by R package “survival”. Bayesian regression was conducted to calculate the coefficients of the genes and selected the candidate genes. Bayesian regression was conducted by R package “spBayesSurv” (Zhang et al., 2019). Finally, principal component analysis (PCA) was conducted to identify the tumor heterogeneity related genes. The tumor neoantigen and stemness based immunotherapy resistance score (TSIRS) was determined as follows:

$$TSIRS=\overset{n}{\underset{i=1}{\sum }}e_{i}*c_{i}$$

$e_{i}$ represents the expression level of the selected genes. $c_{i}$ represents the coefficient of the selected genes.

### Pharmacogenomic analysis

The transcription data of cancer cell lines and the targets of the anti-tumor drugs were downloaded from Genomics of Drug Sensitivity in Cancer (GDSC, http://www.cancerrxgene.org/downloads) (Yang et al., 2013). Spearman correlation analysis was conducted to analyze the correlation between our TSIRS and drug sensitivity. Spearman correlation was used to analyze the correlation between IC50 and TSIRS. *p* < 0.05 was considered statistically significant.

### Cell cultural and CCK-8 assay

BGC-823 cells were cultured with Dulbecco’s Modified Eagle’s Medium (HyClone, UT, USA) containing 10% FBS (Excell Bio, Taicang, China) and penicillin (Beyotime, Shanghai, China) at 37°C and 5% CO_2_. The cells were treated with different doses of PF-4708671 (MedChemExpress, Shanghai, China) for 48 h. The viability of BGC-823 cell was detected using CCK-8 assay kit (Beyotime, Shanghai, China). The procedure followed the manufacturer’s instructions. The OD450 value was detected.

### EdU assay

The proliferation of BGC-823 cells was detected using EdU Kit (RiboBio, Guangzhou, China). The cells were incubated in EdU culture medium (50 μM) for 2 h, fixed with paraformaldehyde (4%), and penetrated with Triton X-100 (0.5%). The nucleus was stained by DAPI reagent.

### Wound healing assay

BGC-823 cells in each group were treated with or without PF-4708671 and then administered with mitomycin for 1 h to inhibit cell proliferation. The wounds were created using a pipette tip. The cells were washed by PBS and then the cultural medium was added. The images were taken at 0, 24 and 48 h after scratching.

### Statistical analysis

Continuous variables between the two groups were compared by Wilcoxon rank sum. The prognosis of patients in the two groups was compared by Log-rank test. TimeROC analysis was conducted to analyze the efficiency of the prediction indexes. The correlation of two continuous variables was analyzed by the Spearman correlation analysis. For the analysis of cell-line assay, the difference between two groups was compared using student-t test. If not specially mentioned, *p* < 0.05 was considered as statistically significant.

## Results

### Characteristics of tumor microenvironment in the response and non-response groups

**FIGURE 1:**
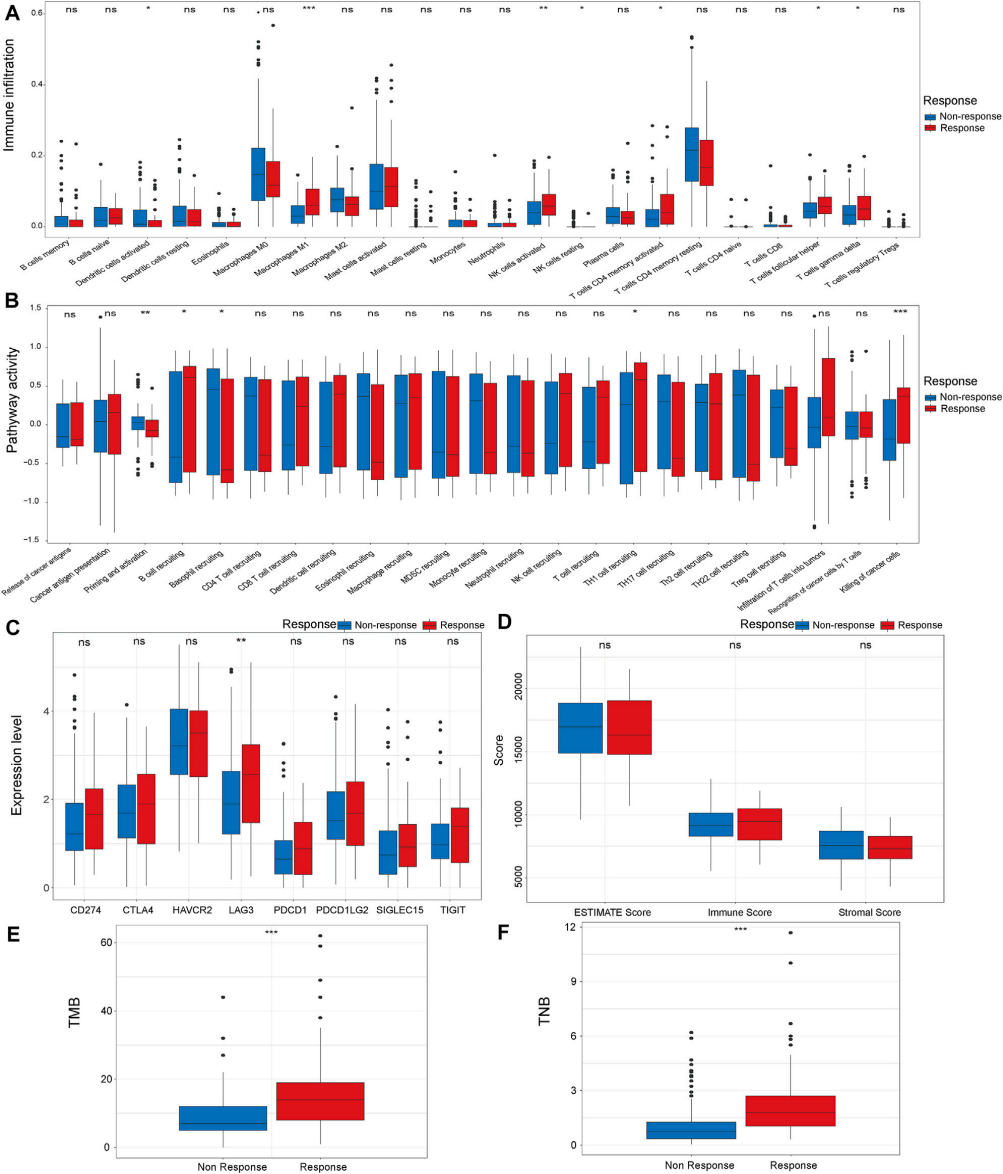
Comparison of microenvironment characteristics of the response and non-response group in IMvigor210 cohort, The immune infiltration level **(A)**, cancer immune circle related process activity **(B)**, immune checkpoint expression **(C)**, ESTIMATE score **(D)**, TMB **(E)** and TNB **(F)** in the response and non-response groups. *p < 0.05, **p < 0.01, ***p < 0.001. TMB: Tumor mutation burden. TNB: Tumor neoantigen burden.

Tumor microenvironment plays a pivotal role in the effectiveness of the immunotherapy (Tomaszewski et al., 2019; Vitale et al., 2019). Thus, we analyzed the microenvironment features of the response and non-response groups. First, we compared the infiltration levels of immune cells in the tumor microenvironment in the response group and non-response group. We found significantly increased levels of M1 macrophages, activated NK cells, activated CD4 memory cells, follicular helper T cells and gamma delta T cells in the immunotherapy response group (Figure 1A). When comparing the activity of cancer immune circle related process in two groups, we found that the activity of the “Killing of cancer cells” process was significantly higher in the response group than in the non-response group (Figure 1B). Next, we analyzed the expression level of immune checkpoints in the two groups. The results demonstrated that the response group has a higher expression level of LAG3 than the non-response group (Figure 1C). ESTIMATE algorithm was applied to evaluate the immune status of the two groups. However, the two groups did not show significant differences in Immune score, Stromal score, and ESTIMATE score (Figure 1D). We also analyzed the somatic mutation features of the two groups. TMB and TNB were significantly higher in the response group compared with the non-response group (Figures 1E,F).

### Construction of the TSIRS

**FIGURE 2:**
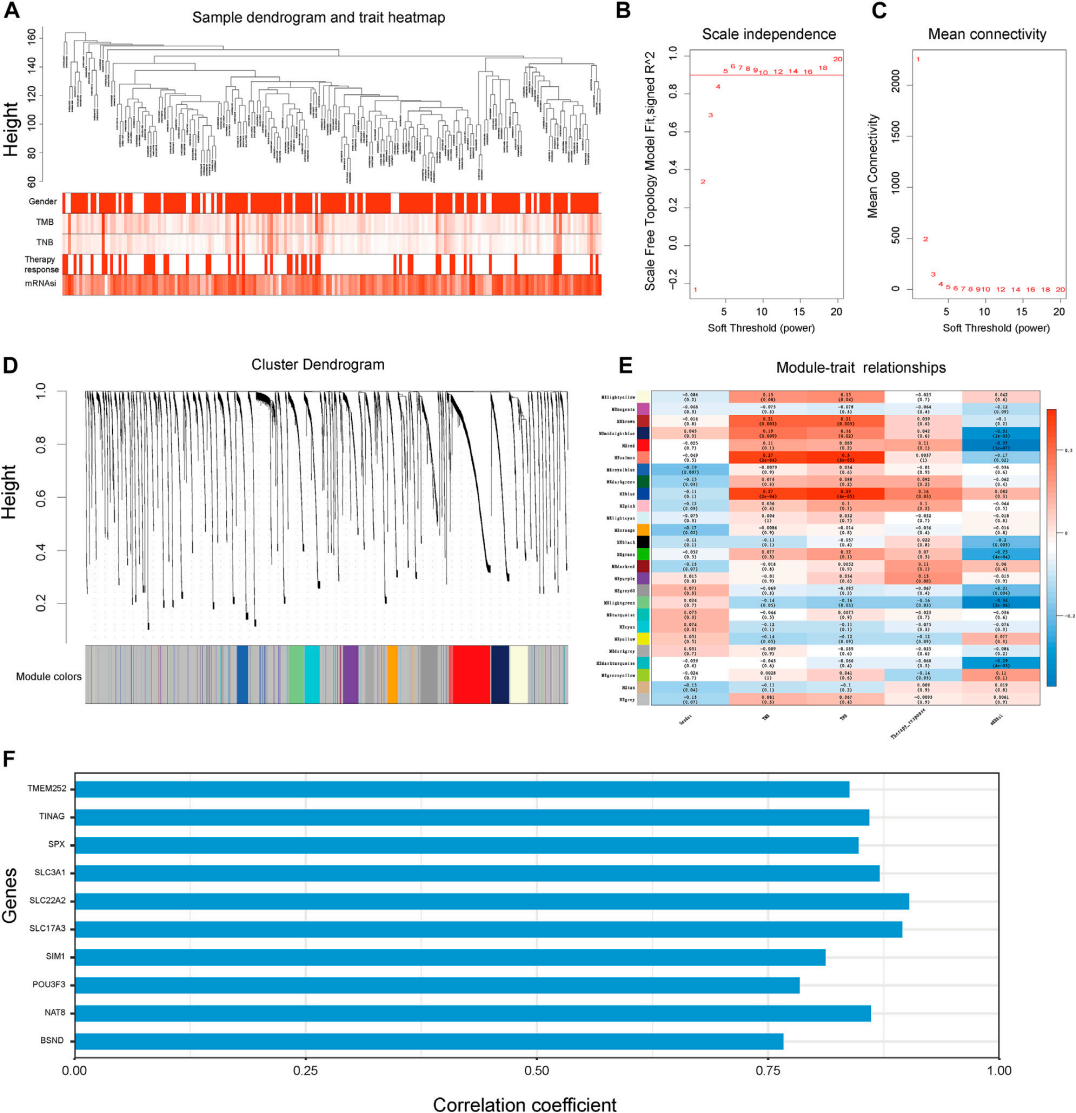
WGCNA analysis to identify the genes module correlated with cancer TNB and mRNAsi for TSIRS construction. **(A)** Clustering dendrogram of samples. **(B)** Scale-free index for different soft-thresholds. **(C)** Mean connectivity for different soft-thresholds. **(D)** Clustering dendrogram of modules. **(E)** Heatmap of the correlation between eigengene and gender, TMB, TNB, therapy responsiveness and mRNAsi. **(F)** Coefficient of selected genes. TMB, Tumor mutation burden; TNB, Tumor neoantigen burden; mRNAsi, mRNA stemness index.

First, we conducted the WGCNA analysis to identify the gene modules correlated with TNB and stemness of cancer. Sample clustering tree was shown in Figure 2A. Then, a network was constructed with a soft threshold of 5 (Figures 2B,C). Next, we built the adjacency matrix and constructed the TOM (Figure 2D). Finally, a total of 26 modules were identified. Among all the 26 modules, module “MElightgreen” was significantly correlated with cancer TNB, mRNAsi and therapy responsiveness (Figure 2E). Thus, genes in module “MElightgreen” were selected for the construction of TSIRS. Candidate genes for TSIRS construction in MElightgreen module were identified and their coefficients were calculated by using a machine-learning-based method (Figure 2F). The TSIRS was determined as the sum of the product of gene expression level and coefficient.

### Enrichment analysis of the module component genes

**FIGURE 3:**
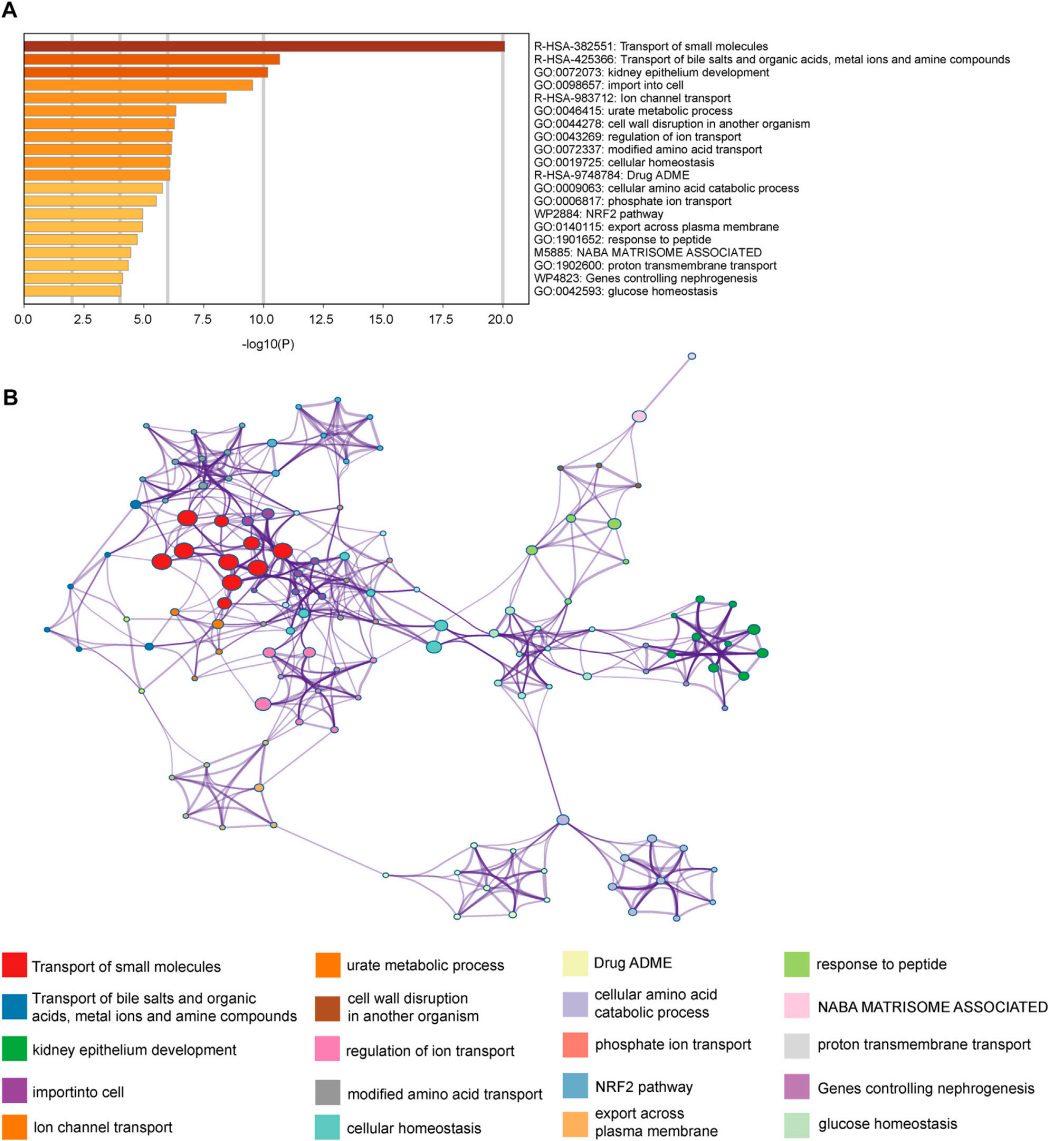
Enrichment analysis revealed the biology functions mediated by module component genes. **(A)** Enrichment terms of the MElightgreen module component genes. **(B)** Network of the enriched pathways.

To investigate the biological function medicated by the genes of MElightgreen module, we applied enrichment analysis *via* Metascape (Figure 3A). The top 3 enriched terms were “transport of small molecules”, “transport of bile salts and organic acids, metal ions and amine compounds” and “kidney epithelium development”. Our results indicated that genes in the module were enriched in the biology function of substance transport (Figure 3B). Meanwhile, the results also indicated that the enriched terms were enriched in the “Transport of the small molecules” (Figure 3B). It implied that the small molecules metabolic features of cancer may play an essential role in the immunotherapy resistance.

### Heterogeneity between the high TSIRS group and low TSIRS group

**FIGURE 4:**
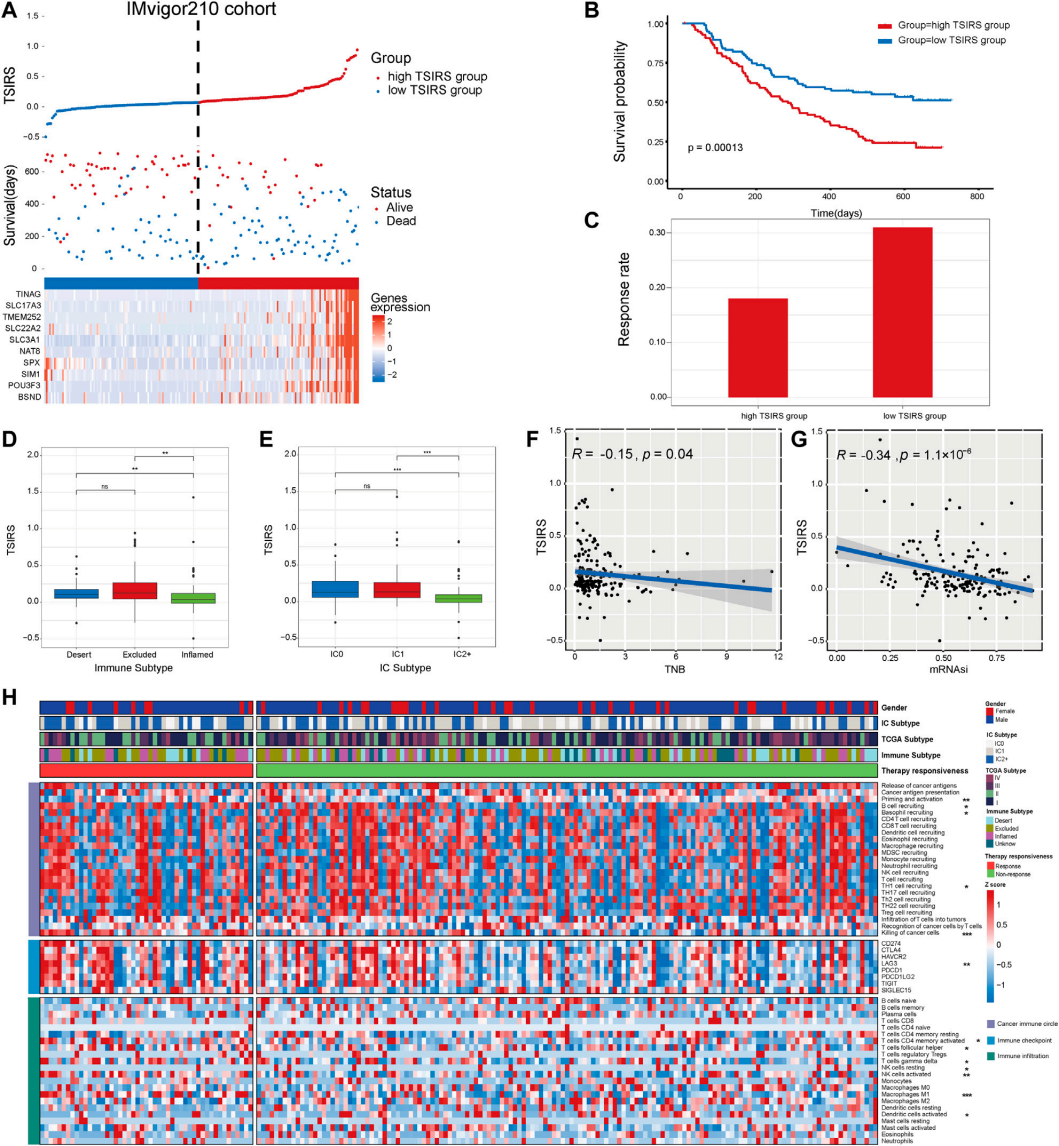
TSIRS predicts patient prognosis in the training cohort. **(A)** Distribution of the high TSIRS group and the low TSIRS group in IMvigor210cohort. **(B)** Comparison of patient prognosis of the high TSIRS group and the low TSIRS group in IMvigor210 cohort. **(C)** Patient therapy responsiveness rate of the high TSIRS group and the low TSIRS group in IMvigor210 cohort. **(D,E)** Comparison of patient TSIRS in different immune **(D)** and IC **(E)** subtype. **(F,G)** Correlation between TSIRS and TNB **(F)** and mRNAsi **(G)**. **(H)** Comparison of patient cancer immune circle related process activity, immune checkpoint expression and immune infiltration level. *p < 0.05, **p < 0.01, ***p < 0.001. TNB, Tumor neoantigen burden; mRNAsi, mRNA stemness index.

First, we calculated the TSIRS in the training cohort. The patients were divided into the high TSIRS group and low TSIRS group (Figure 4A). We found that the prognosis was significantly worse in the high TSIRS group (Figure 4B). The drug responsiveness rate was also lower in the high TSIRS group (Figure 4C). Next, we compared the TSIRS in samples of different immune and IC subtypes. Among the 3 immune subtypes, immune inflamed subtype has the lowest TSIRS (Figure 4D). Meanwhile, IC2+ subtype was lowest TSIRS (Figure 4E). Spearman correlation analysis indicated that TSIRS was negatively correlated with cancer TNB and mRNAsi (Figures 4F,G). These results also indicated that the TSIRS can well predict the responsiveness of immunotherapy. Next, we compared the microenvironment characteristics between the high TSIRS group and low TSIRS group (Figure 4H). Microenvironment characteristics were significantly different in the high TSIRS group and low TSIRS group, including cancer immune circle related process activity, immune checkpoint expression, and immune infiltration.

### TSIRS well predicts patient prognosis and therapy responsiveness in validation cohorts

**FIGURE 5:**
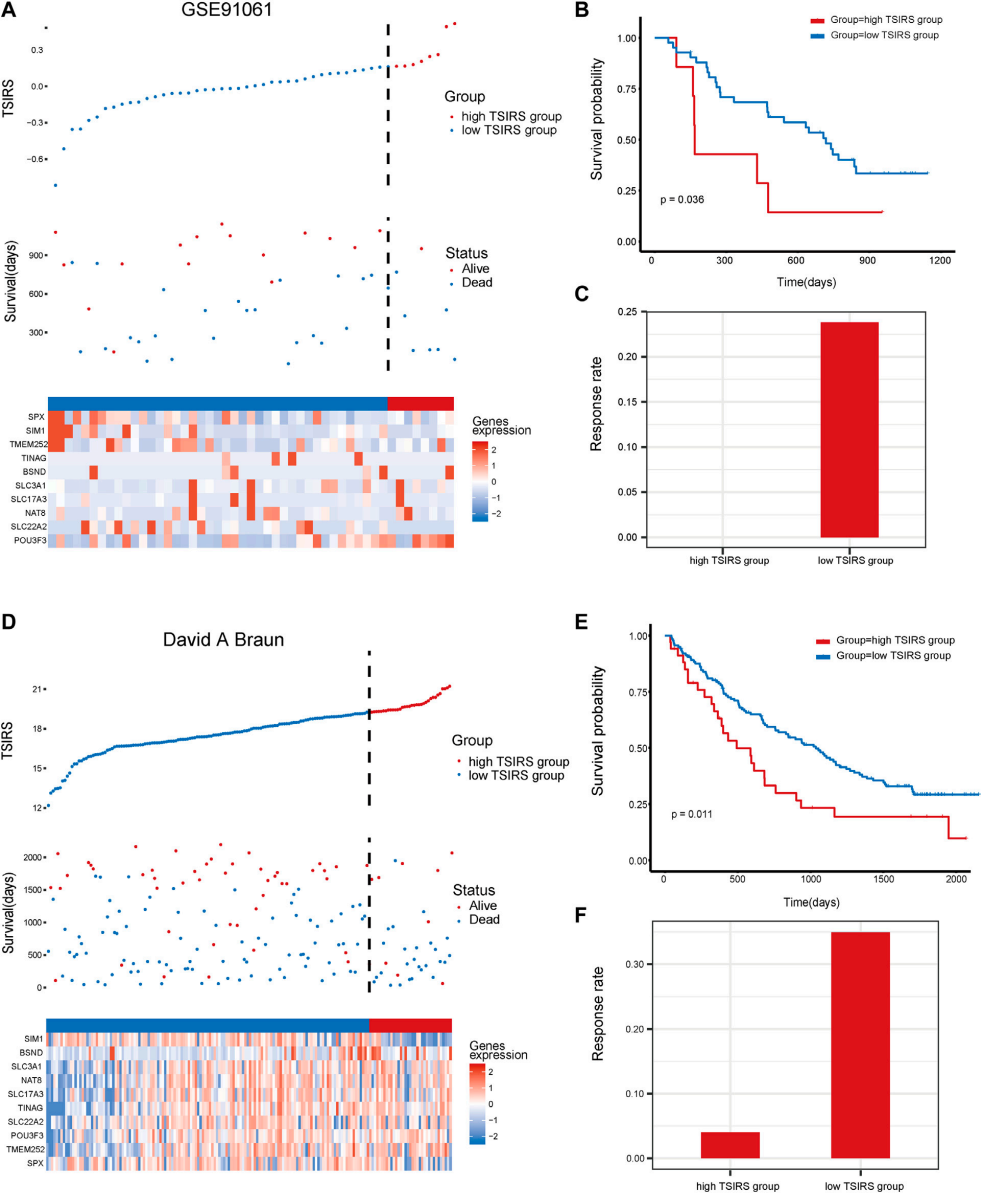
The prognosis effectiveness of TSIRS in GSE91061 cohort and David A. Braun cohort. **(A)** Distribution of the high TSIRS group and the low TSIRS group in GSE91061 cohort. **(B)** Comparison of patient prognosis of the high TSIRS group and the low TSIRS group in GSE91061 cohort. **(C)** Patient therapy responsiveness rate of the high TSIRS group and the low TSIRS group in GSE91061 cohort. **(D)** Distribution of the high TSIRS group and the low TSIRS group in David A. Braun cohort. **(E)** Comparison of patient prognosis of the high TSIRS group and the low TSIRS group in David A. Braun cohort. **(F)** Patient therapy responsiveness rate of the high TSIRS group and the low TSIRS group in David A. Braun cohort.

To further test the robustness of the TSIRS in predicting anti-PD1/PDL1 therapy responsiveness, we calculated the TSIRS of samples in GSE91061 cohort and David A. Braun cohort. The samples of GSE91061 cohort and David A. Braun cohort were classified into the high TSIRS group and the low TSIRS group, respectively (Figures 5A,D). In the two external validation cohorts, the high TSIRS group has the significantly worse prognosis compared to the low TSIRS group (Figures 5B,E). Furthermore, the therapy responsiveness rate of the high TSIRS group was lower than the low TSIRS group in the two validation cohorts (Figures 5C,F). Therefore, the TSIRS has high efficiency in predicting the patient outcome during the anti-PD1/PDL1 therapy.

### TSIRS is a robust tool in predicting responsiveness and cancer immune features during the anti-PD1/PDL1 therapy

**FIGURE 6:**
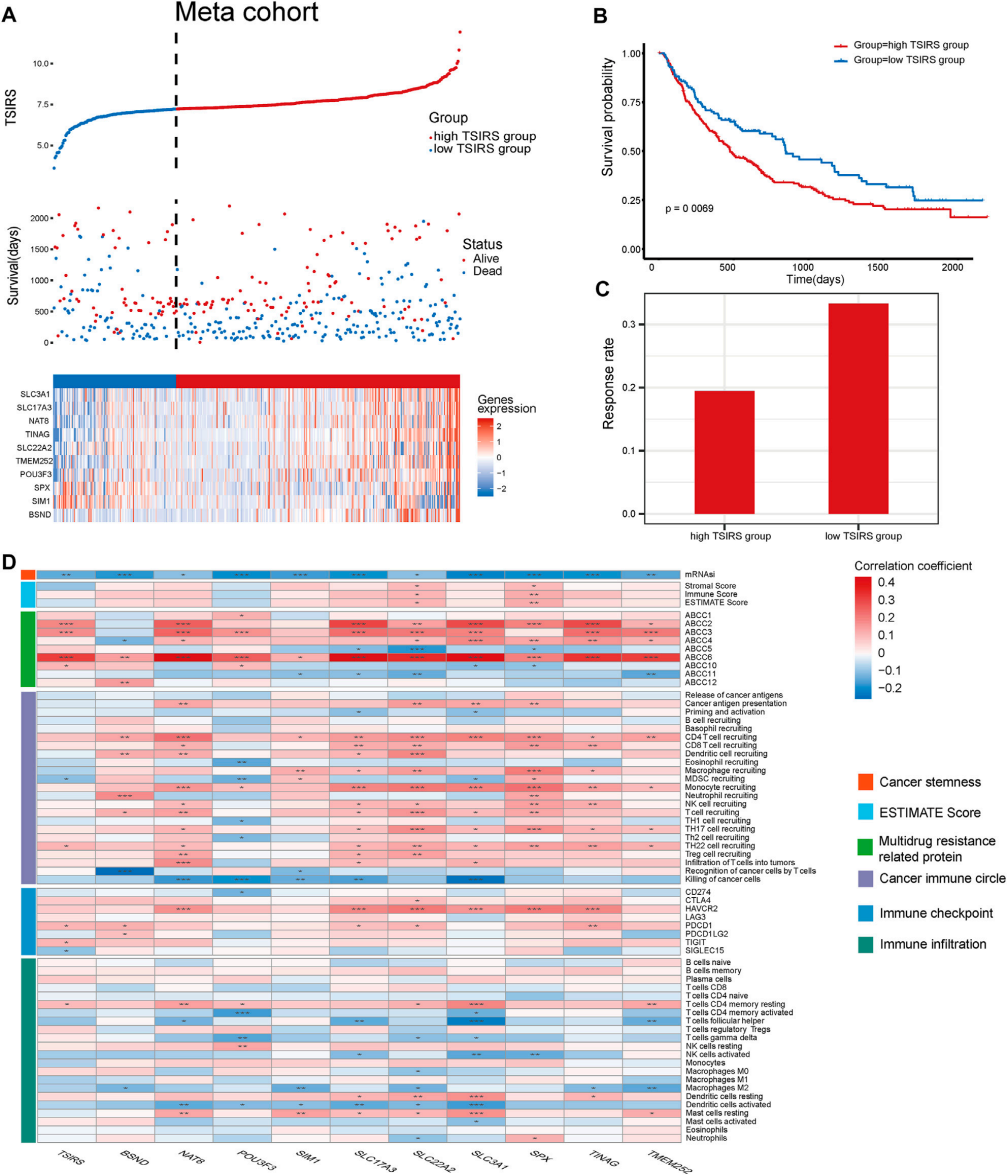
TSIRS predicts prognosis and microenvironment characteristics of patients during anti-PD1/PDL1 treatment, **(A)** Distribution of the high TSIRS group and the low TSIRS group in meta cohort. **(B)** Comparison of patient prognosis of the high TSIRS group and the low TSIRS group in meta cohort. **(C)** Patient therapy responsiveness rate of the high TSIRS group and the low TSIRS group in meta cohort. **(D)** Correlation between TSIRS and cancer stemness, ESTIMATE Score, expression level of genes coding multidrug resistance related protein, cancer immune circle related pathway activity, immune checkpoint expression and immune infiltration level. *p < 0.05, **p < 0.01, ***p < 0.001.

We then constructed the meta cohort based on the three anti-PD1/PDL1 therapy cohorts. TSIRS of the meta cohort samples was calculated. Next, meta cohort samples were divided into high TSIRS and the low TSIRS group according to the calculated scores (Figure 6A). Our result demonstrated that patients with high TSIRS have significantly worse prognosis compared with low TSIRS (Figure 6B). Meanwhile, high TSIRS patients have a lower therapy responsiveness rate (Figure 6C). The result further indicated the robustness of the TSIRS in predicting the responsiveness and prognosis of patients with anti-PD1/PDL1 therapy. We then analyzed the correlation between the TSIRS and cancer microenvironment features (Figure 6D). We found that the TSIRS was negatively correlated with cancer stemness (mRNAsi) and positively correlated with the expression of multi-drug resistance associated protein (ABCC2, ABCC3, ABCC6 and ABCC10). This result suggested that patients with resistance to anti-PD1/PDL1 therapy may have potential multi-drug resistance characteristics.

### The identification of potential alternative drugs for patients resistant to anti-PD1/PDL1 therapy

**FIGURE 7:**
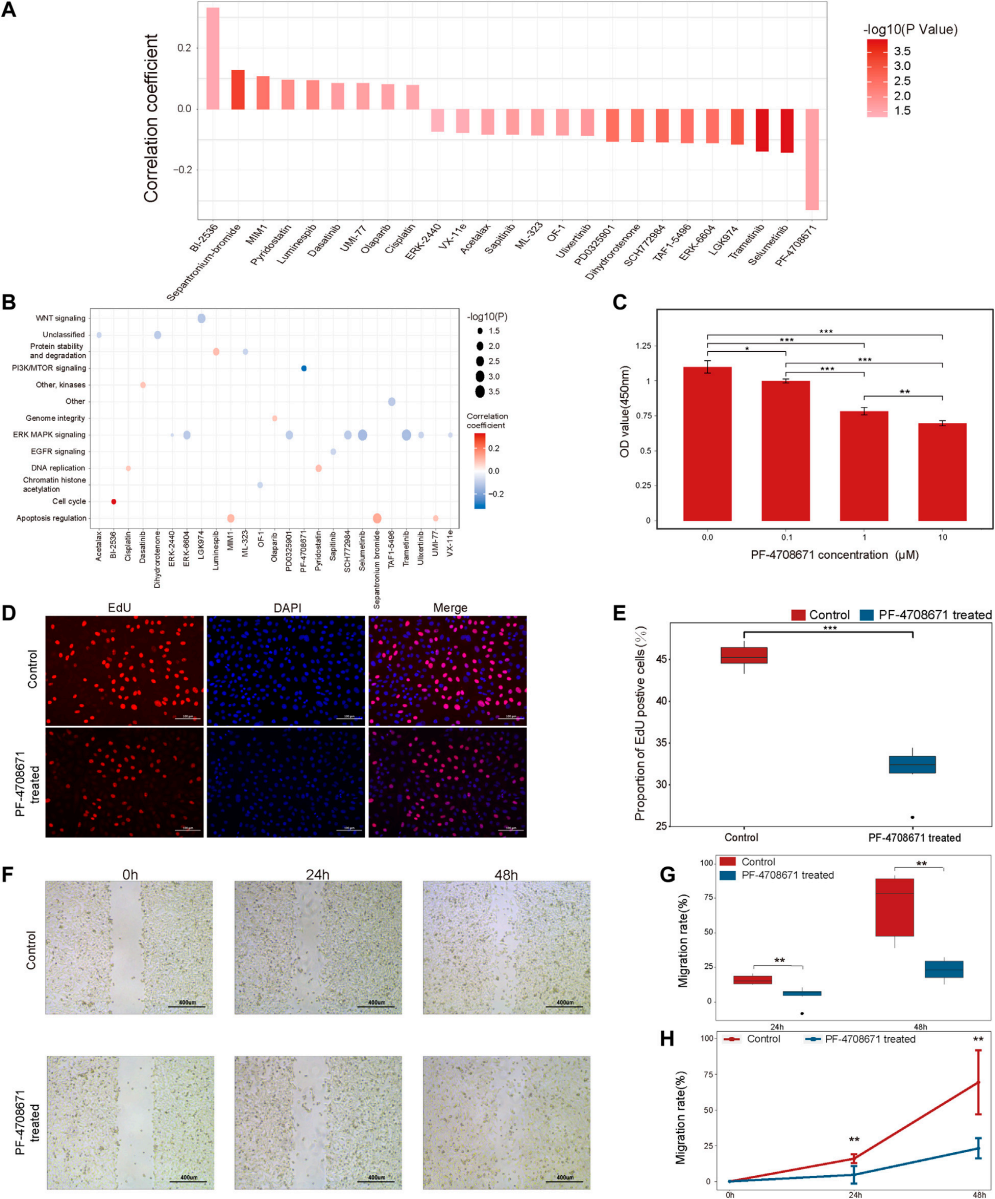
The potential alternative drug for patients resistant to anti-PD1/PDL1 therapy, **(A)** The correlation between the TSIRS and drug IC50 value. **(B)** Targeting pathways of the drugs. **(C)** The effect of PF-4708671 on the viability of cancer cells. **(D)** Representative images of EdU staining. Scale bar: 100 μm. **(E)** The effect of PF-4708671 on the proliferation ability of cancer cells. *n* = 6. **(F)** Representative images of wound healing assay. Scale bar: 400 μm. **(G,H)** The effect of PF-4708671 in cancer cells presented as the box plot **(G)** and line chart **(H)**. *n* = 6. *p < 0.05, **p < 0.01, ***p < 0.001.

To explore the potential alternative drugs for immunotherapy resistant patients, we analyzed the correlation between TSIRS and drug responsiveness in cancer cells. By spearman correlation analysis, we identified 25 drugs whose sensitivity is correlated with TSIRS (Figure 7A). The targets of the 25 identified drugs were shown in Figure 7B. Among all the selected drugs, the IC50 of PF-4708671 has the lowest correlation coefficient with TSIRS, which suggests that PF-4708671 may be the potential alternative for patients resistant to anti-PD1/PDL1 therapy. Thus, PF-4708671 was selected for further analysis. CCK-8 assay, EdU assay and wound healing assay were conducted to validate the anti-cancer efficiency of PF-4708671. The Results indicated that PF-4708671 can effectively inhibit the proliferation and migration of cancer cells (Figures 7C–H).

## Discussion

Immunotherapy, especially anti-PD1/PDL1 therapy, is an important component of current comprehensive cancer treatment (Cramer et al., 2019; Ralli et al., 2020). Many clinical trials have demonstrated that anti-PD1/PDL1 therapy can achieve satisfactory clinical benefits for solid tumors (Robert et al., 2015; Schmid et al., 2018). However, the high non-response rate of anti-PD1/PDL1 therapy is a major application problem (Rosenbaum et al., 2021). The underlying immune mechanism of non-responsive to anti-PD1/PDL1 therapy is also not fully understood. Thus, we developed a robust tool for predicting anti-PD1/PDL1 therapy response and analyzed the immune characteristics potentially associated with anti-PD1/PDL1 therapy responsiveness.

First, we analyzed the immune heterogeneity across the immunotherapy response group and non-response group. We found that the response group has a higher immune activity, TNB and TMB than that in the non-response group. Furthermore, the expression level of immune checkpoint LAG3 is higher in the response group. Recent studies demonstrated that high LAG3 expression associates with the immune suppressive microenvironment (Andrews et al., 2017). Meanwhile, LAG3 is a promising cancer therapy target (Ruffo et al., 2019). These microenvironment differences between the response group and non-response group may contribute to the distinct clinical outcome of the two groups and inferred potential therapy resistance mechanism.

TNB and cancer stemness are two important indicators for predicting the responsiveness of immunotherapy (Wang P. et al., 2021; Unver, 2021). Thus, WGCNA was applied to identify the gene modules correlated with TNB and cancer stemness. In order to predict the responsiveness of patients to anti-PD1/PDL1 therapy, a gene model was constructed based on the identified gene modules using multiple machine-learning methods. When comparing the biological features of the high TSIRS group and the low TSIRS group, we found that infiltration level of M1 microphage was significantly different in the two groups. M1 microphage is the macrophage with a pro-inflammatory phenotype that plays an important role in maintaining anti-tumor immune response (Najafi et al., 2019). This difference in immune microenvironment may contribute to the non-response of the high TSIRS group. In the validation cohorts, TSIRS also effectively predicted the prognosis of patients with anti-PD1/PDL1 therapy. Thus, TSIRS can be a potential tool for the identification of anti-PD1/PDL1 therapy candidates in clinic practice.

We also explored the potential of TSIRS in predicting the characteristics of the cancer immune microenvironment. Among the 9 multidrug resistance related proteins, TSIRS was significantly positive correlated with the expression level of 4 of them. The ABC protein family is the transport proteins which is the driver of drug efflux across the cell membrane (Wang P. et al., 2021). They play important roles in multidrug resistance of cancer (Jadhao et al., 2021). Thus, the anti-PD1/PDL1 therapy resistant cancer patients may exhibit multidrug resistance. Meanwhile, TSIRS was significantly correlated with the infiltration level of CD4 T cells (memory resting) and expression level of TIGIT. It has been found that TIGIT can enhance the activity of Treg cells and contribute to the formation of tumor immune suppression microenvironment (Chen et al., 2020). The expression of TIGIT may associate with the resting status of CD4 T cells. Thus, TSIRS can well predict immune status of tumor microenvironment.

We next attempted to explore alternative drugs for patients who did not benefit from anti-PD1/PDL1 therapy. Pharmacogenomic analysis and cancer cell-based experiment implied that PF-4708671 may be potentially applied in the patients with high TSIRS. PF-4708671 is a specific inhibitor of p70 ribosomal S6 kinase 1 (Park et al., 2015). Qiu ZX et al. indicated that PF-4708671 has the inhibitory effect on non-small cell lung cancer (Qiu et al., 2016). Our results also revealed that PF-4708671 can inhibit the proliferation and migration of cancer cells. Therefore, PF-4708671 may be an adjuvant for patients with high TSIRS.

The study still has some limitations. First, due to the lack of the publicly available data of anti-PD1/PDL1 therapy, the validation based on the data from immunotherapy cohorts remains inadequate. Second, the in-depth *in vitro* and *in vivo* experiment are needed to further validate the application potential and anti-cancer mechanism of the predicted drugs. Further biological studies are in demand to explore the clinic translation potential of PF-4708671. These deficiencies will be improved with the progress of immunotherapy-related big data and the further studies.

In conclusion, our study provides a robust anti-PD1/PDL1 therapy resistance prediction tool-TSIRS based on the TNB and stemness of cancer. The TSIRS can well predict the prognosis and microenvironment features of patients receiving anti-PD1/PDL1 treatment and has great application potential in precision cancer therapy. For patients with resistance to anti-PD1/PDL1 therapy, PF-4708671 may be developed as a candidate for cancer comprehensive treatment.

## Data Availability

The datasets presented in this study can be found in online repositories. The names of the repository/repositories and accession number(s) can be found in the article.
